# Poly(2-oxazoline)/saRNA Polyplexes for Targeted and Nonviral Gene Delivery

**DOI:** 10.1021/acs.biomac.3c00683

**Published:** 2023-10-04

**Authors:** Graham Hayes, Beatriz Dias-Barbieri, Gokhan Yilmaz, Robin J. Shattock, C. Remzi Becer

**Affiliations:** ‡ Department of Chemistry, University of Warwick, Coventry, CV4 7AL, United Kingdom; § Department of Infectious Diseases, Imperial College London, Norfolk Place, London W21PG, United Kingdom

## Abstract

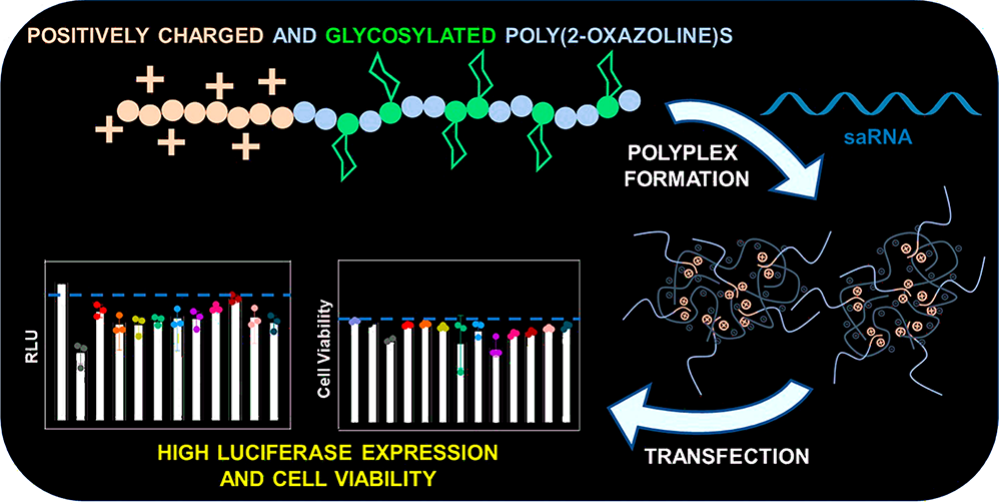

RNA delivery has been demonstrated to be a potent method of vaccine delivery, as demonstrated by the recent success of the COVID-19 vaccines. Polymers have been shown to be effective vehicles for RNA delivery, with poly(ethylene imine) (PEI) being the current gold standard for delivery. Nonetheless, PEI has toxicity concerns, and so finding alternatives is desirable. Poly(2-oxazoline)s are a promising alternative to PEI, as they are generally biocompatible and offer a high degree of control over the polymer structure. Here, we have synthesized an ionizable primary amine 2-oxazoline and combined it with a double bond containing oxazoline to synthesize a small library of charged statistical and block copolymers. The pendant double bonds were reacted further to decorate the polymers with glucose via a thiol–ene click reaction. All polymers were shown to have excellent cell viability, and the synthesized block polymers showed promising complexation efficiencies for the saRNA, demonstrating a clear structure–property relationship. The polymer transfection potential was tested in various cell lines, and a polymer composition with an amine/glucose ratio of 9:27 has demonstrated the best transfection potential across all cell lines tested. Overall, the results suggest that block polymers with a cationic segment and high levels of glycosylation have the best complexation efficiency and RNA expression levels.

## 1 Introduction

Gene delivery is one of the most novel and exciting techniques for the conveyance of therapeutics and vaccinations at present. For example, RNA vaccines were used extensively against the SARS-CoV-2 outbreak with great effect.^1^ Theoretically, RNA vaccines could be used to provide protection against a wide range of infectious diseases including influenza,^2^ rabies,^3^ HIV,^4^ and Ebola.^5^ RNA vaccines have several advantages over traditional vaccines that use the direct injection of antigens or deactivated viruses. Traditional vaccines require large-scale bioreactors that grow batches of cells that then produce the virus or antigen protein, which is costly and time-consuming. For RNA vaccines, RNA is produced synthetically and then combined with a delivery vehicle. LNPs are typically used to deliver RNA vaccines, although the stability and storage of using LNPs as delivery agents can be problematic.^6^ Another issue is that the general population can experience side effects upon exposure to the RNA vaccines due to an innate ability to detect RNA.^7^ These side effects are directly correlated to the amount of RNA used in the vaccine, so minimizing the amount of RNA injected is of current interest. One method of reducing the payload of RNA required is to use self-amplifying RNA (saRNA). As well as encoding the antigen, saRNA also encodes a replicase protein that can replicate the original strand of injected RNA, and thus amplify protein expression.^8^ Nonetheless, one of the problems with saRNA is that it is much larger than mRNA and is more difficult to deliver.^9^ Several different delivery vehicles have been used to deliver RNA effectively, including LNPs,^10^ cationic polymers,^11^ dendrimers,^12^ and nanofiber-type materials.^13^

Regarding polyplexes, various polymers have been extensively studied including poly(ethylene imine) (PEI),^14^ poly(2-oxazoline)s,^15^ poly(ethylene glycol),^16^ and peptides.^17^ Among the different polymers tested, PEI is generally viewed as the optimal transfection agent.^18^ PEI is generally synthesized via either the ring opening polymerization of aziridine,^19^ or the hydrolysis of linear poly(2-ethyl-2-oxazoline)s.^20^ Nonetheless, both of these methods have the disadvantage of an associated lack of control, which results in uncertainty about the exact polymeric structure. Partially hydrolyzed poly(2-oxazoline)s have also been used for RNA delivery,^21^ but again, this method is imprecise and does not allow for the formation of complex structures such as defined, functionalized, cationic block polymers. Therefore, access to these well controlled cationic poly(2-oxazoline) structures would allow for comparison of different architectures such as block polymers and statistical polymers. Poly(2-oxazoline)s are an ideal polymer class for this type of application due to their versatility and biocompatibility, as well as their peptoid structure.^22,23^ Indeed, complex poly(2-oxazoline) architectures such as graft copolymers have previously been demonstrated to selectively target different types of liver cell.^24^

Lectins are proteins that regulate biological processes such as cell recognition and intracellular communication.^25,26^ They achieve this by binding glycans such as oligosaccharides on the surface of cells and viruses, and play an important role in human disease.^27^ Since lectins bind sugars, glycopolymers can be used to target specific cells for applications such as drug delivery. Indeed, the type of sugar used and its spatial configuration in relationship to the polymer backbone have been shown to be able to influence the lectin selectivity.^28^ Furthermore, poly(2-oxazoline)s decorated with sugar moieties have been demonstrated to be effective at targeting specific cells.^29^ Nonetheless, the synthetic route used in this case limits the polymer architecture to random copolymers, which reduces the polymer definition and could impact lectin selectivity. Therefore, a method combining charged poly(2-oxazoline)s with sugars in a manner that enables structures such as blocks is highly desirable. As mentioned, many poly(2-oxazoline)s are regarded as being biocompatible and this is one of the main reasons for the intensive research into them currently.^30−32^ They are also generally water-soluble and exhibit stealth-like behavior in the body, meaning they can circulate in the body while remaining undetected by the immune system.^24,33^

**Scheme 1:**
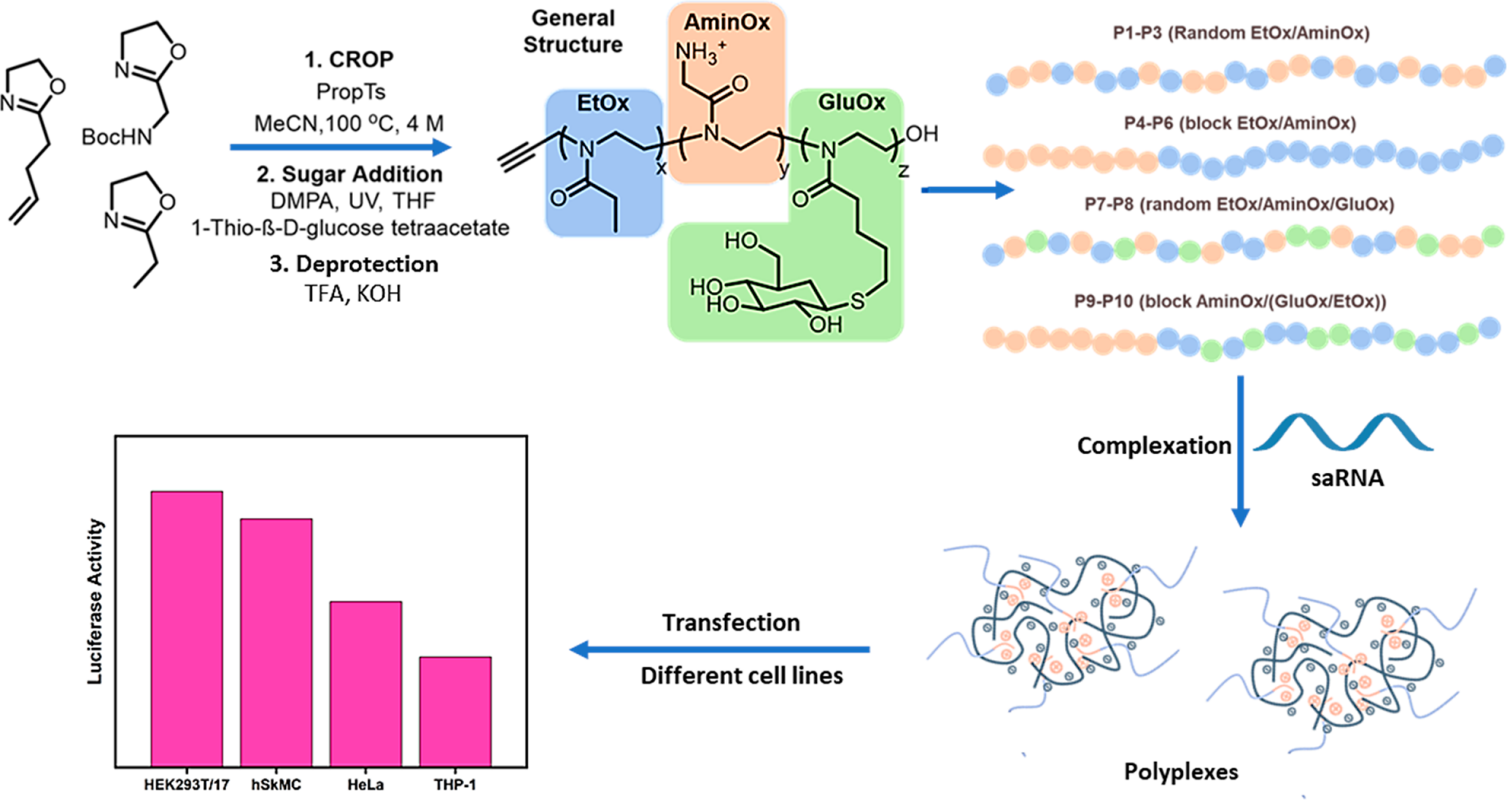
Schematic Illustration of the Synthesis of Glycosylated Cationic Poly(2-oxazoline)s in Different Structures and the Formed Polyplexes with saRNA for Transfection Experiments in Different Cell Lines by Luciferase Assay

Herein, a library of poly(2-oxazoline)s has been synthesized by combining three discrete monomer types: 2-ethyl-2-oxazoline (EtOx), 2-butenyl-2-oxazoline (butenylOx), and a protected amine oxazoline (BocAmineOx) were combined in various ratios. The butenylOx was then used to attach glucose units via a thiol–ene reaction, which were then deprotected (GluOx), and the BocAmineOx was deprotected to leave a cationic amine (AmineOx) (Scheme 1). Using these monomers, various statistical copolymers and block polymers have been synthesized to study the effect of the polymer structure on saRNA transfection. Each polymer was tested at different N/P ratios to investigate the best ratio for transfection, before comparing their complexation efficiency, transfection efficiency, and toxicity in various cell lines, including HEK293T/17, HeLa, hSkMC, and THP-1.

## 2 Experimental Section

### 2.1 Materials

Boc-glycine (98%), *N*-(3-(dimethylamino)propyl)-*N*′-ethylcarbodiimide (EDAC; 97%), 4-dimethylaminopyridine (DMAP; 99%), 2,2-dimethyl-2-phenyl-acetophenone (DMPA; 99%), 1-thio-β-d-glucose tetraacetate (97%), sodium chloride (>99%), THF (anhydrous), and triethylamine (>99%) were purchased from Sigma-Aldrich and used as received. Chloroethylamine·HCl (98%), acetonitrile (anhydrous), and hydrochloric acid (37%) were purchased from Fisher Scientific and used as received. Trifluoroacetic acid (99%) and 4-pentenoic acid (98%) were purchased from Alfa-Aesar and used as received. Methyl *p*-toluenesulfonate (98%; Fisher Scientific) was distilled prior to use. 2-Ethyl-2-oxazoline (99%; Fisher Scientific) was stirred over calcium hydride for 16 h before purification by distillation.

### 2.2 Analytical Techniques

#### Nuclear Magnetic Resonance (^1^H NMR and ^13^C NMR)

All spectra were recorded on a Bruker Avance III HD 400 MHz. CDCl_3_ was used as solvent, and the signal of the residual CHCl_3_ served as reference for the chemical shift, δ. The data analysis was performed by using TopSpin 3.2 software.

#### Gel Permeation Chromatography (GPC)

The eluent used was THF with 2% TEA and 0.01% BHT. The Agilent Technologies 1260 Infinity instrument was equipped with a refractive index (RI) and 308 nm UV detector, a PLgel 5 μm guard column, and a PLgel 5 μm mixed D column (300 × 7.5 mm). Samples were run at 1 mL min^–1^ at 40 °C. Poly(methyl methacrylate) standards (Agilent PMMA calibration kits M-M-10 and M-L-10) were used for the calibration. Before injection (100 μL), the samples were filtered through a PTFE membrane with a 0.2 μL pore size.

#### Dynamic Light Scattering (DLS)

DLS measurements were performed by using a Malvern μV Zetasizer equipped with an 830 nm laser and a scattering angle of 90° at a temperature of 20 °C. Samples were filtered with a 0.4 μm PVDF-filter (Whatman) to prevent the presence of dust.

#### Cell Transfection and Luciferase Assay

The cell experiments were carried out via the following procedure: Transfection assay was performed similar to as previously described by Blakney et al.^2^ For both HEK293T/17 and HeLa cell lines, a concentration of 5 × 10^4^ cells per well was seeded in a 96-well plate 24 h prior to the experiment. For THP-1 cells, the concentration was 8 × 10^4^ cells per well, and for the immortalized hSkMC, the concentration was 10 × 10^4^ cells per well. On the day of the experiment, 100 ng of polyplexes in 100 μL of ultrapure H_2_O was added to each well. Samples were allowed to transfect for 24 h. After, the transfection efficiency was analyzed by removing 50 μL of medium and adding 50 μL of Bright-Glo luciferin substrate (Promega, U.K.) into each well. The total volume was transferred to a white plate (Falcon, U.S.), and the luminescence intensity was analyzed on a FLUOstar Omega plate reader (BMG Labtech, U.K.).

#### Quantification of Complexation Efficiency

The saRNA payload in polyplexes was quantified using a Quant-iT RiboGreen assay (Thermo Fisher, U.K.) similar to that previously described.^37^ Samples were diluted to 3 μg/mL in 1× TE buffer in PBS (Sigma-Aldrich, U.K.). Standard solutions were also prepared in a 1× TE buffer to account for any variation in fluorescence. The assay was performed according to the manufacturer’s protocol. Ribogreen reagent was diluted 200-fold in 1× TE buffer. Samples were loaded on a black, 96-well plate and analyzed for fluorescence on a microplate reader (BMG Labtech, U.K.) at an excitation of 485 nm and emission at 528 nm. Fluorescence values correspond to the RNA that was not loaded into polyplexes, and the percentage of saRNA loading was calculated by subtracting it from 100%. The experiment was replicated on two occasions.

#### Cell Viability Assay

Cells were seeded at the appropriate concentrations, as mentioned previously, and transfected the next day with 100 ng of RNA complexed with polymers. Cells were incubated with polyplexes for 24 h. Plates were equilibrated at room temperature for 30 min, and an equal volume of the CellTiter-Glo 2.0 (Promega, U.K.) reagent was added to the wells (100 μL). Contents were mixed for 2 min using an orbital shaker, and plates were incubated for 10 min at room temperature. The total volume was transferred to a white plate (Falcon, U.S.), and luminescence intensity was analyzed on a FLUOstar Omega plate reader (BMG Labtech, U.K.).

#### In Vitro Transcription of Self-Amplifying mRNA

Self-amplifying mRNA (saRNA) derived from VEEV alphavirus genome and encoding firefly luciferase (fLuc) was prepared by in vitro transcription. pDNA was linearized using MluI (New England BioLabs, U.K.) for 2 h at 37 °C; MluI was added again and incubated for another 1 h at 37 °C. Linearization was confirmed by agarose gel electrophoresis. For transcription into saRNA, 6 μL of linearized DNA template was synthesized into RNA transcripts via the mMessage mMachine kit (Invitrogen, Thermo Fisher Scientific, U.K.). Transcripts were then purified by lithium chloride (LiCl) precipitation. Briefly, transcripts were frozen overnight at −20 °C and precipitated the next morning by centrifugation at 14000 rpm for 20 min at 4 °C. Pellets were resuspended in 70% ethanol and centrifuged at 14000 rpm for 5 min at 4 °C. The ethanol was removed, pellets were allowed to dry for 5 min, and transcripts were resuspended in ultrapure H_2_O. RNA quantification was done using a NanoDrop One (Thermo Fisher Scientific, U.K.) and RNA integrity was evaluated by RNA gel electrophoresis using a FlashGel System (Lonza, U.K.).

#### Formulation of Polyplexes

Stock solutions of the polymers (**P1**–**P10**) at 1 mg/mL were prepared in ultrapure H_2_O. Polyplexes were prepared at different N/P ratios (0.5, 1, 5, 20, and 50). The required amount of polymer at different N/P ratios was added to a fixed amount of RNA (20 μg). Polymers were added in a dropwise manner to the RNA solution in HEPES buffer with 5% glucose (pH 5). Samples were mixed for 30 min at 500 rpm and at 20 °C, using a Thermomixer (Eppendorf, Germany).

#### Cell Line and Culture Conditions

HEK293T.17 and HeLa cells (ATCC, U.S.) were routinely grown in Dulbecco’s Modified Eagle’s Medium (DMEM; Gibco, Thermo Fisher, U.K.) supplemented with 10% (v/v) fetal bovine serum (FBS), 1% (v/v) l-glutamine, and 1% (v/v) penicillin/streptomycin (Thermo Fisher, U.K.), at 37 °C under 5% CO_2_. When confluent, cells were washed with DPBS 1× (Gibco, U.K.) and treated with trypsin (TrypLE Express 1×; Gibco, U.K.) for seeding in new culture flasks (Corning, U.S.). THP-1 cells (ATCC, U.S.) were routinely grown in RPMI-1640 Medium (Sigma, U.K.) supplemented with 10% (v/v) fetal bovine serum (FBS), 1% (v/v) l-glutamine, and 1% (v/v) penicillin/streptomycin (Thermo Fisher, U.K.), at 37 °C under 5% CO_2_. When confluent, the whole cell suspension in culture media was centrifuged at 1750 rpm for 5 min, and the pellet was resuspended in fresh RPMI-1640 medium for seeding in new culture flasks (Corning, U.S.). Finally, an immortalized cell line of human skeletal muscle cells (hSkMC; PromoCell, U.K.) was routinely grown in Skeletal Muscle Cell Growth Medium (PromoCell, Germany) supplemented with SupplementMix (PromoCell, Germany). When confluent, cells were washed with DPBS 1× (Gibco, U.K.) and treated with trypsin. Neutralization was done with DPBS 1× containing 10% FBS, and cells were centrifuged and resuspended in the skeletal muscle cell growth medium for seeding in new culture flasks (Corning, U.S.).

### 2.3 Synthesis

#### Synthesis of BocAmineOx

To a 500 mL round-bottomed flask were added *N*-(*tert*-butoxycarbonyl) glycine (10.00 g, 57.1 mmol, 1 equiv), chloroethylamine·HCl (7.28 g, 62.8 mmol, 1.1 equiv), and DMAP (0.697 g, 5.7 mmol, 0.1 equiv), along with a magnetic stirrer bar. DCM (200 mL) was added, and the reaction was stirred in a flask under a nitrogen blanket and cooled to 0 °C using an ice bath. Once the reaction mixture had cooled to 0 °C, triethylamine (11.55 g, 110 mmol, 2 equiv) was added dropwise. Next, *N*-(3-(dimethylamino)propyl)-*N*′-ethylcarbodiimide (EDAC; 9.74 g, 62.8 mmol, 1.1 equiv) was added dropwise, and the reaction mixture was allowed to stir overnight. Next, the reaction mixture was washed with 0.5 M HCl_(aq)_ (3 × 100 mL) saturated NaHCO_3_ solution (3 × 100 mL), distilled water (3 × 100 mL), and brine (2 × 100 mL) before being dried over magnesium sulfate. The solvent was removed in vacuo to yield the amide intermediate. For the ring closure step to form the 2-oxazoline, potassium hydroxide (4.8 g, 85 mmol, 1.5 equiv) was dissolved in methanol (50 mL). The amide intermediate was placed in a 100 mL round-bottomed flask with a stirrer bar and placed under a nitrogen blanket. To this, the methanolic potassium hydroxide solution was added slowly, and the reaction mixture was then heated to 50 °C and left for 16 h. The reaction mixture was then filtered, and the residual methanol was removed in vacuo. The reaction mixture was then redissolved in DCM (100 mL) before being washed with distilled water (3 × 100 mL) and then brine (2 × 100 mL) before being dried over magnesium sulfate. The organic solvent was removed in vacuo before the 2-oxazoline was purified by vacuum distillation to yield a white crystalline solid (overall yield = 70%). NMR spectra can be found in the Supporting Information, Figures S2 and S3.

^1^H NMR (400 MHz, CDCl_3_): δ 5.10 (s, 1H), 4.32 (t, *J* = 9.74 Hz, 2H), 3.95 (d, *J* = 4.80 Hz, 2H), 3.85 (t, *J* = 9.74 Hz, 2H), 1.45 (s, 9H). ^13^C NMR (400 MHz, CDCl_3_, DEPT) δ CH_2_, 68.2; CH_2_, 54.1; CH_2_, 38.0; CH_3_, 28.3

#### Synthesis of ButenylOx

To a 500 mL round-bottomed flask were added 4-pentenoic acid (10.00 g, 99.8 mmol, 1 equiv), chloroethylamine·HCl (12.74 g, 109.9 mmol, 1.1 equiv), and DMAP (1.22 g, 10.0 mmol, 0.1 equiv), along with a magnetic stirrer bar. DCM (200 mL) was added, and the reaction flask was stirred under a nitrogen blanket and cooled to 0 °C using an ice bath. Once the reaction mixture had cooled to 0 °C, triethylamine (20.2 g, 199.9 mmol, 2 equiv) was added dropwise. Next, *N*-(3-(dimethylamino)propyl)-*N*′-ethylcarbodiimide (EDAC; 17.06 g, 109.9 mmol, 1.1 equiv) was added dropwise, and the reaction mixture was allowed to stir overnight. Next, the reaction mixture was washed with 0.5 M HCl_(aq)_ (3 × 100 mL) saturated NaHCO_3_ solution (3 × 100 mL), distilled water (3 × 100 mL), and brine (2 × 100 mL) before being dried over magnesium sulfate. The solvent was removed in vacuo to yield the intermediated amide. For the ring closure step to form the 2-oxazoline, potassium hydroxide (8.4 g, 149.8 mmol, 1.5 equiv) was dissolved in methanol (50 mL). The amide intermediate was added to a 100 mL round-bottomed flask with a stirrer bar and placed under a nitrogen blanket. To this, the methanolic potassium hydroxide solution was added slowly, and the reaction mixture was then heated to 50 °C and left for 16 h. The reaction mixture was then filtered, and the residual methanol was removed in vacuo. The reaction mixture was then redissolved in DCM (100 mL) before being washed with distilled water (3 × 100 mL) and then brine (2 × 100 mL) before being dried over magnesium sulfate. The organic solvent was removed in vacuo, before the 2-oxazoline was purified by vacuum distillation to yield a white crystalline solid (overall yield = 40%). NMR spectra can be found in the Supporting Information, Figures S4 and S5.

^1^H NMR (400 MHz, CDCl_3_) δ 5.84 (m, 1H), 5.04 (m, 2H), 4.22 (t, *J* = 9.23 Hz, 2H), 3.82 (t, *J* = 9.23 Hz, 2H), 2.38 (s, 4H). ^13^C NMR (400 MHz, CDCl_3_, DEPT) δ C, 167.7; CH, 136.8; CH_2_, 115.3; CH_2_, 67.1; CH_2_, 54.3; CH_2_, 29.8; CH_2_, 27.3.

#### Synthesis of Random Poly(2-oxazoline) Copolymer (**P7**)

To a clean and dry microwave vial, bocAmineOx (0.16 g, 0.8 mmol, 10 equiv) was added with a stirrer bar, before the flask was sealed and placed under a nitrogen atmosphere. To this were added butenylOx (0.10 g, 0.8 mmol, 10 equiv), 2-ethyl-2-oxazoline (0.39 g, 0.40 mL, 4.0 mmol, 50 equiv), and acetonitrile (0.75 mL) were added. The reaction mixture was then degassed with nitrogen for 10 min, before methyl *p*-toluenesulfonate (14.8 mg, 12.0 μL, 0.0799 mmol, 1 equiv) was added. A sample was taken for *t*_0_ before the reaction was placed in an oil bath at 100 °C for 100 min. Next, a sample was taken for *T*_final_ before the polymer was precipitated twice in diethyl ether. The same procedure was followed for polymers **P1**, **P2**, **P3**, and **P8**. Quantities of reagents, reaction times, and monomer conversions can be seen in Table S1.

#### Synthesis of p(EtOx)-p(BocAmineOx) Block Poly(2-oxazoline)s (**P4**)

To a clean and dry microwave vial, 2-ethyl-2-oxazoline (0.495 g, 4.99 mmol, 40 equiv) and acetonitrile (0.75 mL) were added. The reaction mixture was then degassed with nitrogen for 10 min before propargyl *p*-toluenesulfonate (26.0 mg, 0.12 mmol, 1 equiv) was added. A sample was taken for *t*_0_ before the reaction was placed in an oil bath at 100 °C for 75 min. After this time, bocAmineOx (0.250 mg, 1.24 mmol, 10 equiv) was added to the reaction flask. The reaction flask was then left for a further 25 min at 100 °C. Next, a sample was taken for *T*_final_ before the polymer was precipitated twice in diethyl ether. The polymer was then deprotected (see Deprotection of BocAmineOx). The same procedure was followed for polymers **P5** and **P6**. Quantities of reagents, reaction times, and monomer conversions can be seen in Table S1.

#### Synthesis of (p(EtOx)-r-p(GluOx))-*b*-p(BocAmineOx) Block Poly(2-oxazoline)s (**P9**)

To a clean and dry microwave vial, 2-ethyl-2-oxazoline (0.495 g, 4.99 mmol, 40 equiv), butenylOx (0.100 g, 0.08 mmol, 10 equiv), and acetonitrile (0.75 mL) were added. The reaction mixture was then degassed with nitrogen for 10 min, before methyl *p*-toluenesulfonate (15.0 mg, 0.08 mmol, 1 equiv) was added. A sample was taken for *t*_0_ before the reaction was placed in an oil bath at 100 °C for 100 min. After this time, bocAmineOx (0.160 mg, 0.8 mmol, 10 equiv) was added to the reaction flask. The reaction flask was then left for a further 20 min at 100 °C. Next, a sample was taken for *T*_final_ before the polymer was precipitated twice in diethyl ether. The polymer then underwent postpolymerization to add the glucose (see the glycosylation step), before deprotection of the bocAmineOx and then deprotection of the glucose. The same procedure was followed for polymer **P10**. Quantities of reagents, reaction times, and monomer conversions can be seen in Table S1.

#### Deprotection of BocAmineOx

The protected polymer was dissolved in DCM (2 mL) and trifluoroacetic acid (1 mL) was added to the reaction mixture. The mixture was left to stir overnight at room temperature, before the polymer was precipitated in diethyl ether, and subsequently dried in vacuo before being dialyzed against a 0.5 M sodium chloride solution using 1 kDa cutoff dialysis tubing.

#### Addition and Deprotection of Thioglucose to Polymer Chains

Polymer **P9** (100 mg), 2,2-dimethoxy-2-phenylacetophenone (45 mg, 0.5 equiv per butenylOx), and 1-thio-β-d-glucose tetraacetate (250 mg, 2 equiv per butenylOx) were dissolved in dry THF (0.75 mL). The reaction mixture was stirred under UV radiation for 16 h before precipitation of the polymer in diethyl ether. For glucose deprotection, polymer **P9** (50 mg) was dissolved in methanol (2.5 mL). To this, 2 M sodium methoxide in methanol (0.5 mL) was added, and the reaction mixture was stirred at room temperature for 3 h before the addition of 1 M HCl to obtain a reaction mixture pH of ∼3. The polymer was then precipitated in diethyl ether and subsequently dialyzed against 0.5 M NaCl solution using 1 kDa cutoff dialysis tubing. Quantities of DMPA and 1-thio-β-d-glucose tetraacetate used can be found in Table S2.

## 3 Results and Discussion

### 3.1 Synthesis of Cationically Charged Poly(2-oxazoline)s

**Figure 1:**
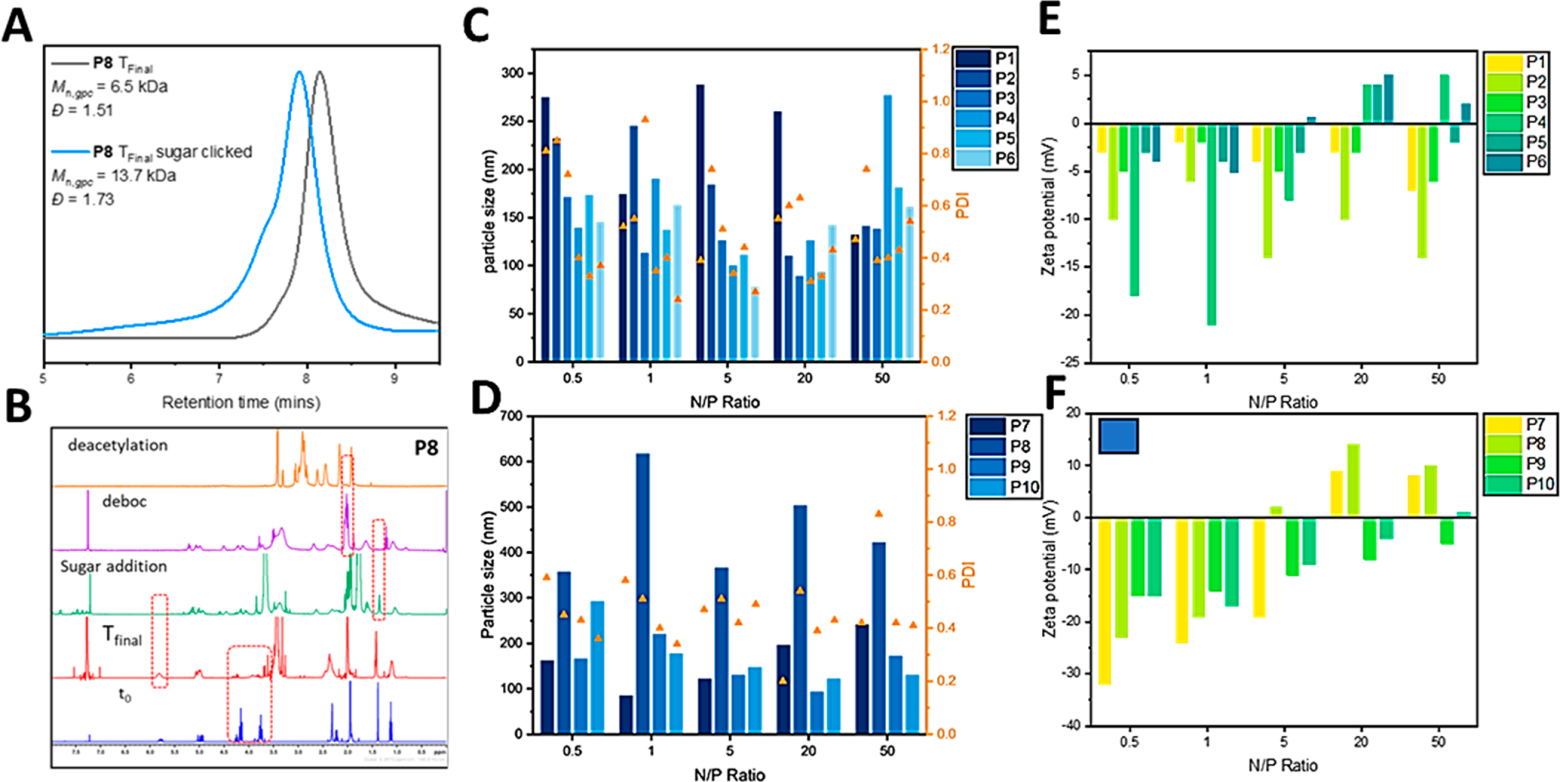
(A) GPC traces before and after click reaction of **P8** with sugar; (B) ^1^H NMR results of each step of the synthesis of **P8**; (C) Particle size and PDI of **P1**–**P6** polyplexes with saRNA; (D) Particle size and PDI of **P7**–**P10** polyplexes with saRNA; (E) Zeta potential of **P1**–**P6** polyplexes with saRNA; and (F) Zeta potential of **P7**–**P10** polyplexes with saRNA.

Cationic ring-opening polymerization (CROP) was used to polymerize 2-oxazoline based monomers. As shown in Table 1, different types of cationic poly(2-oxazoline)s have been prepared successfully with low dispersity and high molecular weight. Basically, EtOx monomer was combined with an ionizable primary amine and a double bond 2-oxazoline monomer to synthesize a small library of charged statistical and block copolymers. The pendant double bonds were reacted further to decorate the polymers with sugar via a thiol–ene click reaction. **P1**–**P3** are statistical copolymers of various compositions between EtOx and AmineOx. **P4**–**P6** are block polymers of various lengths between EtOx and AmineOx. **P7** and **P8** are random polymers among EtOx, GluOx, and AmineOx. Finally, **P9** and **P10** are block polymers, with the first block being a random combination of EtOx and GluOx and the second block being purely AmineOx. Glycosylated polymers exhibited elevated dispersity compared to other polymers due to the presence of high molecular weight shoulders in the GPC traces (Figure 1A). As depicted in Figure 1B, the progress of monomer conversion was monitored through ^1^H NMR analysis, focusing on the oxazoline ring signals located at 3.8 and 4.2 ppm. The peaks corresponding to the alkene groups, which initially appeared in the range of 5.8–6.0 ppm, completely disappeared after the thiol–ene reaction. Simultaneously, new signals emerged at around 2.0 ppm, signifying the presence of acetyl protecting groups on the sugars. This peak of the acetate groups disappeared in the ^1^H NMR spectra after deprotection of the obtained polymers in MeOH with sodium methoxide for 3 h at room temperature.

**Table 1:** Summary of Polymers Used for Transfection Studies, Along with Their Monomer Conversions, and Number Average Molar Masses (*M*_n(GPC)_) and Molar Mass Distributions (*Đ*)

entry	type	EtOx conv.^a^	BocAmineOx conv.^a^	ButenylOx conv. (DP)^a^	*M*_n(GPC)_^b^ (kDa)	*M*_n(theor.)_ (kDa)	*Đ*^b^	CE^c^ (%)
**P1**	P(EtOx_70_-*r*-AmineOx_11_)	>99%	90%		6.6	9.1	1.31	14
**P2**	P(EtOx_95_-*r*-AmineOx_10_)	>99%	90%		7.3	11.4	1.23	19
**P3**	P(EtOx_90_-*r*-AmineOx_10_)	>99%	>99%		10.1	11.0	1.11	14
**P4**	P(EtOx_40_-*b*-AmineOx_10_)	>99%	>99%		4.9	5.2	1.31	93
**P5**	P(EtOx_60_-*b*-AmineOx_10_)	>99%	>99%		6.2	8.2	1.34	85
**P6**	P(EtOx_80_-*b*-AmineOx_18_)	>99%	87%		10.5	11.7	1.13	95
**P7**	P(EtOx_52_-*r*-AmineOx_10_-*r*-GluOx_9_)	>99%	>99%	90%	13.4	12.0	1.39	31
**P8**	P(EtOx_27_-*r*-AmineOx_9_-*r*-GluOx_27_)	>90%	90%	90%	13.7	17.7	1.73	30
**P9**	P(EtOx_56_/GluOx_9_)-*b*-P(AmineOx_5_)	>99% (56)	60% (5)	>99% (9)	12.6	11.1	1.23	64
**P10**	P(EtOx_53_/GluOx_10_)-*b*-P(AmineOx_10_)	>99% (53)	83% (10)	>99% (10)	15.0	12.2	1.35	93

a Measured by ^1^H NMR.

b Measured by GPC. The eluent used was THF with 2% TEA and 0.01% BHT. The Agilent Technologies 1260 Infinity instrument was equipped with a refractive index (RI) and 308 nm UV detectors, a PLgel 5 μm guard column, and a PLgel 5 μm mixed D column (300 × 7.5 mm). Samples were run at 1 mL min^–1^ at 40 °C. Poly(methyl methacrylate) standards (Agilent PMMA calibration kits, M-M-10 and M-L-10) were used for the calibration. Before injection (100 μL), the samples were filtered through a PTFE membrane with a 0.2 μL pore size.

c Complexation efficiency (CE), from RiboGreen assay. Each polymerization was monitored by ^1^H NMR and was quenched when reaching nearly complete conversion.

### 3.2 Optimizing the N/P Ratio for saRNA Complexation

To discover the best formulation parameters for polyplex formation, the synthesized polymers were first screened across a range of N/P ratios from 0.5 to 50. Initially, the size, PDI, and zeta potentials of each polyplex were measured by DLS (Figure 1C–F). In general, the polyplexes were smaller at higher N/P ratios, and the zeta potentials were much closer to neutral, with some positive polyplexes, as expected. In general, for an N/P ratio of 20, the polyplex sizes were among the smallest with the lowest dispersities, with the zeta potentials showing the most positive results compared to the other ratios. Interestingly, the glycosylated polymers were generally smaller with lower dispersities. One obvious outlier to this trend was **P8**, which although glycosylated, formed very large particles. This unexpected behavior suggests that there might be unique characteristics or factors associated with **P8** that influence its particle size and distribution differently compared to the other glycosylated polymers. It might be because of either the polymer structure in terms of hydrophobicity and positive charge group distribution on the polymer backbone or carbohydrate-carbohydrate interaction between the polyplexes as P8 has the highest amount of sugar units along the polymer backbone.

For the nonglycosylated polymers (**P1**–**P6**), the random copolymers **P1**–**P3** generally formed larger, more disperse particles than the block polymers (**P4**–**P6**). At higher N/P ratios (20,50), the block polymers had higher zeta potentials. At this point, there was no obvious trends between the polymers within the random copolymer subset (**P1**–**P3**), or the polymers within the block polymer subset (**P4**–**P6**). For the glycosylated polymers, the block polymers (**P9** and **P10**) were smaller than the random polymers (**P7** and **P8**), especially at high N/P ratios. **P8** had the highest zeta potential of all of the polymers, formed the largest particles, and had the largest *M*_n_ of all of the polymers tested here. The reason for these features is not clear, but one hypothesis is that the light-induced thiol–ene reaction between the butenylOx double bond and the thiol-glucose has a side reaction that causes polymer–polymer coupling. Indeed, the GPC traces (figure S1) do show a high molecular weight shoulder upon the thiol–ene reaction, and this may be exacerbated for **P8** because it has a larger quantity of double bonds per polymer. **P10** also had higher zeta potential values when compared to **P9**, and this can be attributed to having twice the amount of positively charged units in the polymer (5 per polymer for **P9**, 10 per polymer for **P10**).

Next, the transfection efficiency was measured at each N/P ratio for all of the polymers (see Figure S2) Here, polyplexes were formed using firefly luciferase as a proxy for transfection efficiency as the amount of luminescence emitted can be used to quantify transfection. Again, improved transfection was observed with higher N/P ratios. The nonglycosylated random polymers (**P1**–**P3**) generally performed poorly across the whole N/P ratio, with minimal transfection shown. The block polymers **P4**–**P6** had much higher transfection efficiencies, demonstrating the effect of the polymer architecture on transfection efficiency. The block polymers have a higher concentration of charged species at one terminus of the polymer, as opposed to the random polymers which have the charged species distributed throughout the polymer which explains the improved transfection seen with the block polymers. Within the nonglycosylated block polymers, transfection generally improved with increasing polymer size, with **P6** performing better at low N/P ratios compared to **P5**, and **P5** performing better than **P4** at low N/P ratios. For the random glycosylated polymers, **P8** had improved transfection compared to **P7** at higher N/P ratios, which is likely due to the increasing amount of attached glucoses. Comparing the effect of block polymer vs random polymer for the glycosylated polymers, **P10** performed slightly better than **P7** at lower N/P ratios, but they were similar at higher N/P ratios. Comparison of **P7** and **P10** is important because they have the same quantities of EtOx, AmineOx, and GluOx, but **P10** has the charged AmineOx groups in a block structure while they are randomly distributed for **P7**. Doubling the charged block length from 5 to 10 (**P9** to **P10**) improved transfection at the lower N/P ratios, but at higher N/P ratios (20,50), there was minimal difference. Due to these preliminary transfection results along with the DLS results, an N/P ratio of 20 was selected for future experiments as these polyplexes had the smallest sizes, and had comparable transfection results to an N/P ratio of 50.

### 3.3 Complexation Efficiency of the Polymers

**Figure 2:**
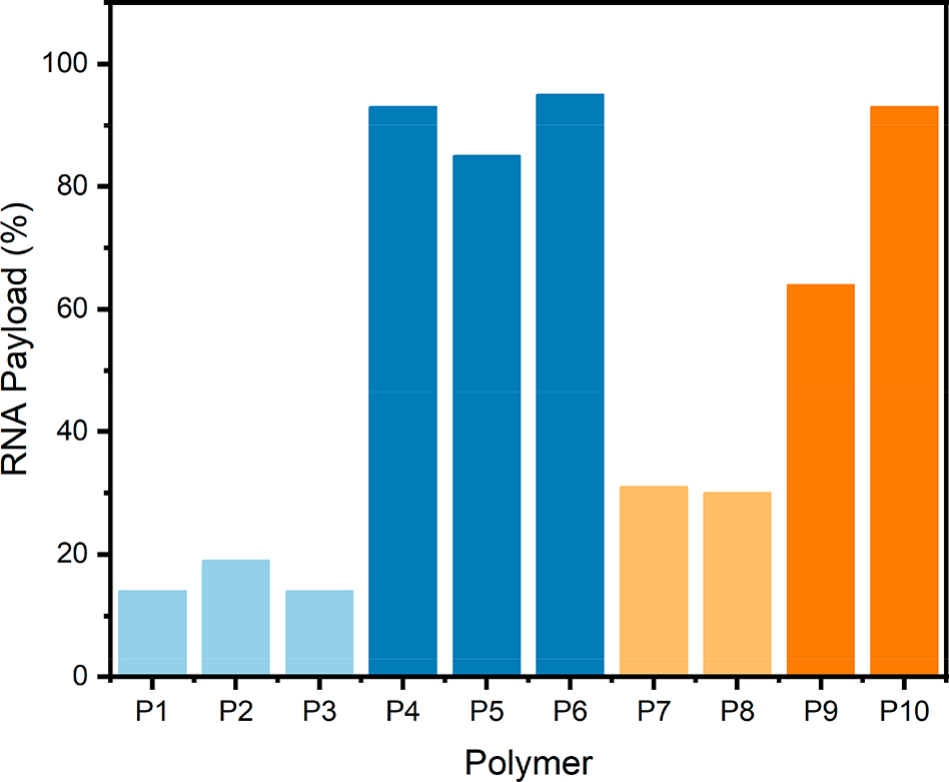
Complexation efficiency results for all of the polymers from the RiboGreen Assay. Colors are provided to aid the reader in distinguishing between the polymer types. Blue polymers are nonglycosylated and orange polymers are glycosylated. Lighter colors are random polymers and darker colors are block polymers.

To measure the complexation efficiency of the polymers, a RiboGreen RNA assay was performed (Figure 2). In this test, a small molecule fluoresces upon binding with RNA, but cannot access RNA that is bound within a polyplex, and so can be used to quantify noncomplexed RNA. Complexation efficiency is important to try to maximize the RNA payload and increase efficacy of any drug system.

For the nonglycosylated random polymers (**P1**–**P3**), the complexation efficiency was low, with the polymer length having only a marginal effect on the complexation efficiency, with the longer chain (**P2**) having slightly improved complexation efficiency compared to **P1** and **P3**. The nonglycosylated block polymers (**P4**–**P6**) showed considerably better complexation efficiency than their random counterparts, with the complexation efficiency increasing to over 80% for all three polymers (see Table 1). The block structure increases the positive charge density at the chain end, and this is likely the reason for the improved transfection efficiency. For the glycosylated polymers, the same trend was seen between the random polymers and the block polymers, i.e. the block polymers **P9** and **P10** performed better than the random polymers **P7** and **P8**. Interestingly, glycosylation appeared to increase the complexation efficiency for the random polymers slightly, as both **P7** and **P8** showed better complexation than **P1**, **P2**, and **P3**. **P10** had slightly improved complexation efficiency compared to **P5**, which has similar DP block lengths with a similar amount of cationic charges but without glycosylation. Lastly, **P9** had a much worse complexation efficiency than **P10**, which is due to the much shorter charged block length (10 units for **P10**, 5 units for **P9**). In order to maximize complexation efficiency, glycosylated block polymers are ideal, with a longer charged block showing improved complexation.

### 3.4 Cell Viability When Exposed to Polyplexes

**Figure 3:**
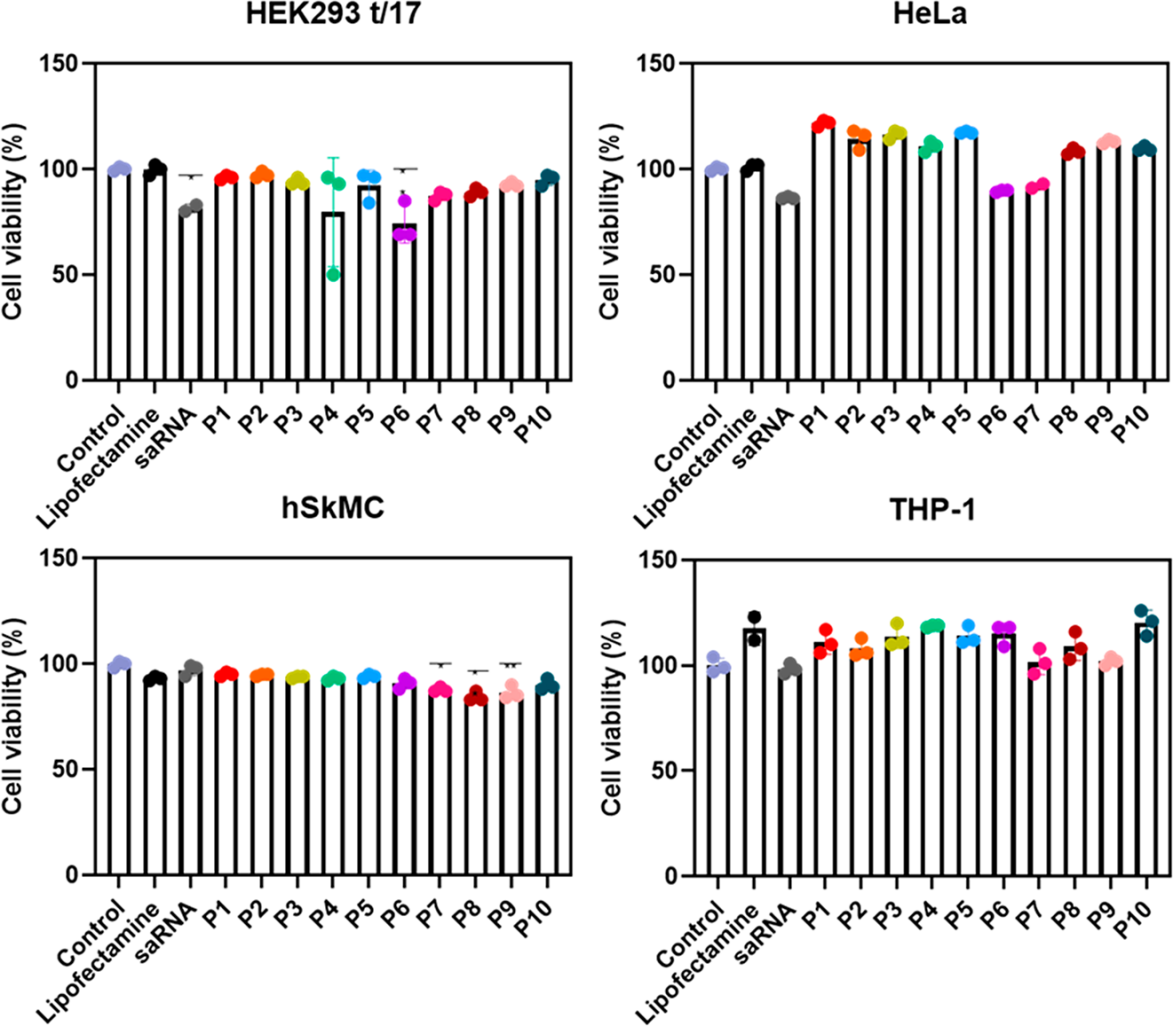
Cell viability of different cell lines upon incubation with the polymers for 24 h, as calculated using the CellTiter-Glo 2.0 assay.

Next, a CellTiter-Glo 2.0 assay test was performed to examine cell viability after the cells had been exposed to the different polyplexes (Figure 3). In general, the cell viability was above 80% for all polymers used in all the cell lines. Polymers **P7** and **P8** showed a small drop in cell viability in the HeLa and HEK293 t/17 cell lines; however, the reduction was not a significant one. For the hSkMC skeletal muscle cell line, polymers **P7**, **P8**, **P9**, and **P10** showed a small drop in cell viability; however, their viability was still above 80% after 24 h. Lastly, all polymers showed excellent viability in the THP-1 cell line. Overall, the cell viability results show that the polymers have excellent compatibility with various different cell types.

### 3.5 Transfection Efficiency in Various Cell Lines

**Figure 4:**
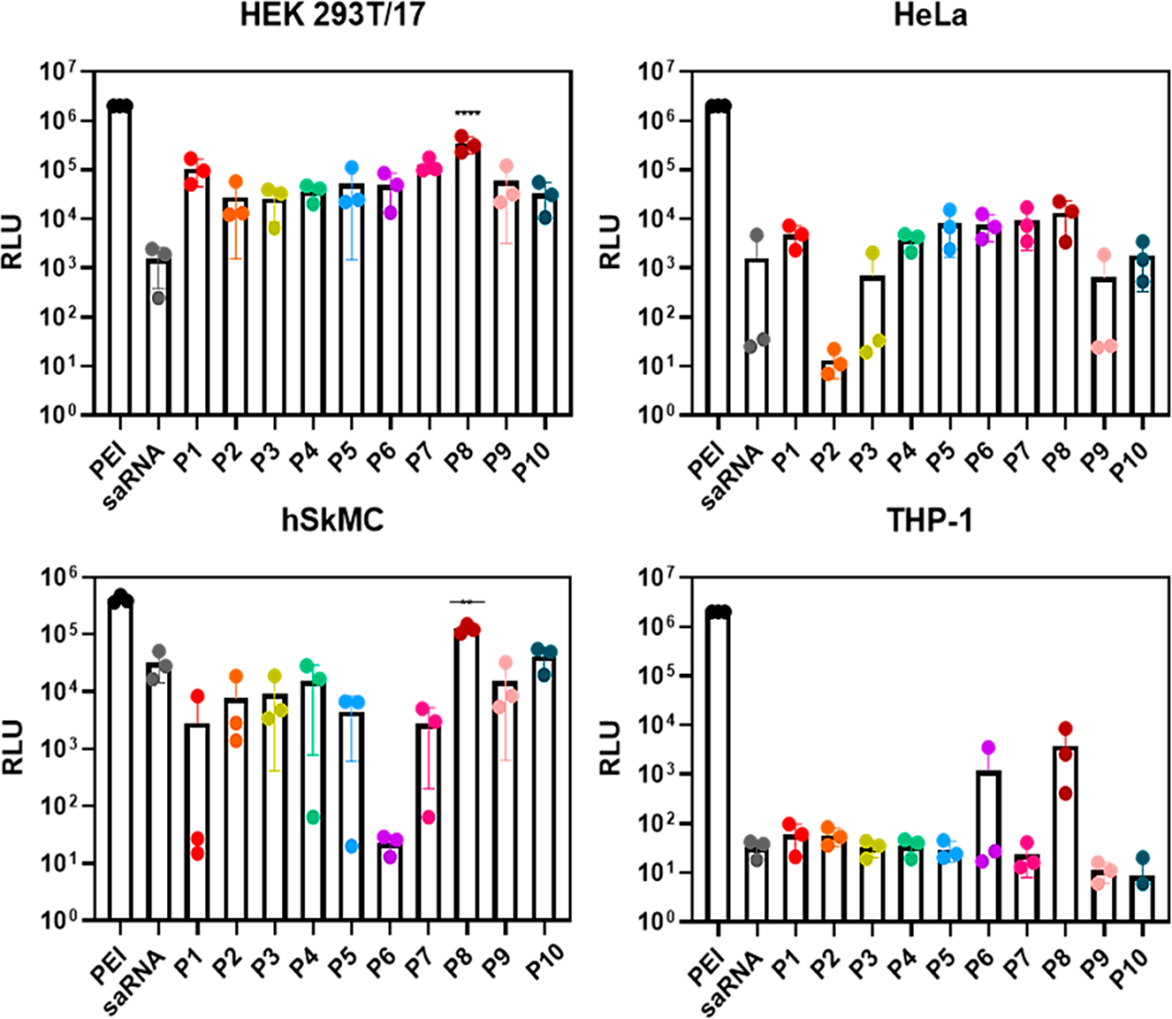
Polyplex efficiency for polymers **P1**–**P10** using cell lines HEK 293T/17, HeLa, hSkMC, and THP-1.

To explore the effect of structure and glycosylation of the polymers on saRNA transfection, transfection efficiency was measured in a variety of cell lines: HEK293T/17, HeLa, hSkMC, and THP-1 (Figure 4). First, the transfection efficiency with the HEK 293T/17 was investigated. Human embryonic kidney (HEK) cells are useful for transfections studies because they are easy to grow and transfect with genes, as they have little regulation on RNA expression.^34^ As expected, all polyplexes for this cell line demonstrated higher transfection efficiency compared to saRNA, although none performed to the same standard as PEI due to the very high molecular weight of PEI compared to the synthesized polymers. For the random nonglycosylated polymers (**P1**–**P3**), increasing the length of the polymer decreased transfection efficiency slightly. This may be due to the reduced concentration of charged units along the backbone for **P2** and **P3** because of the diluting effect of adding more EtOx. For the nonglycosylated block polymers (**P4**–**P6**), increasing the polymer length appeared to have minimal effect, with **P4** and **P6** having largely the same transfection efficiency, despite **P6** having a charged block that is twice the size of **P4**. Perhaps surprisingly, there is minimal difference in transfection efficiency between the nonglycosylated block polymers and the random polymers, despite the large difference in complexation efficiencies. For the glycosylated polymers, the random polymers performed better than the block polymers, with **P8** showing the best transfection efficiency among all the polymers evaluated in the HEK 293T/17 cell line. Interestingly, **P8** also had the highest amount of glycosylation of all the polymers, which could correlate to improving transfection efficiency.

**P7** shows a slight improvement when compared to **P10**, suggesting that using a random copolymer structure shows a better transfection potential compared to a block for the glycosylated polymers. For the glycosylated block polymers, there was little difference in transfection between **P9** and **P10**, suggesting that the charged block length does not affect transfection, corroborating the trend seen with the nonglycosylated block polymers. Overall, for the HEK 293T/17 cell line, all of the polymers performed consistently. The length of the charged block was shown to be less important for transfection. Whether the charged units were distributed throughout the random polymers or concentrated together the block polymers was also shown to be unimportant for the nonglycosylated polymers, but more influential in the glycosylated polymers.

Next, the HeLa cell line was considered, which is an immortalized epithelial cell line. Here, all of the polymers performed worse than PEI, with some appearing to inhibit the transfection of saRNA. The reduction in cell transfection efficiency could be explained by the fact that HeLa cells are interferon (IFN) competent, while HEK cells are not. An excess of type I IFN response may lead to the activation of the eukaryotic translation initiation factor 2A (eIF2A), which impairs the activity of eIF2 and, consequently, inhibits mRNA translation and protein synthesis.^35,36^ The nonglycosylated random polymers performed poorly, with **P1** showing the best transfection, albeit comparative to saRNA on its own. **P2** and **P3** appeared to inhibit saRNA transfection, however the reasons why there is such a large difference between **P1** and **P2**/**3** is not clear, although **P1** is a smaller overall polymer compared to **P2** and **P3**. The nonglycosylated block polymers (**P4**–**P6**) were more consistent; however, there was not a significant difference between the various block lengths.

Nonetheless, **P4** was the smallest block polymer with an *M*_n(theor.)_ of 5.2 kDa and had comparable transfection efficiency to **P1** which had an *M*_n(theor.)_ of 9.1 kDa. For the glycosylated random polymers **P7** and **P8**, addition of glucose did not change the transfection significantly when compared to the nonglycosylated random polymers. This is in contrast with the HEK293*T*/17 cell line, in which the glycosylated random polymers showed improved efficiency when compared to the nonglycosylated polymers. Lastly, the glycosylated block polymers **P9** and **P10** showed a reduction in transfection efficiency when compared to the glycosylated random polymers, although **P10** appeared to show some improvement over **P9** presumably due to the increased length of the charged block. For the HeLa cell line, polymers showed a dramatic reduction in transfection efficiency when they had too high a degree of polymerization and were random polymers. Furthermore, when the amount of charged units was lower than 10 per polymer (**P9**) the transfection efficiency was also was reduced.

Continuing from the HeLa cell line, the hSkMC (human skeletal muscle) cell line was investigated. Generally, all of the polyplexes exhibited lower transfection efficiency compared to saRNA alone, except for **P8** which showed improved transfection efficiency when compared to saRNA and was almost as effective as PEI, which is a promising result considering the difference in molecular weight between PEI (40 kDa) and **P8** (17.7 kDa). Regarding the random nonglycosylated polymers, increasing the polymer length appeared to improve transfection, although all three polymers (**P1**–**P3**) demonstrated lower transfection efficiency compared to saRNA. Interestingly, **P6** showed much lower transfection efficiency than all other polymers for the hSkMC cell line. **P6** contains more charged units than all of the others, which could be the reason for this observation. However, PEI contains a large amount of charged secondary amines along the backbone of the polymer, while retaining high transfection efficiency. Moreover, the polymers in this series contain primary amines and so increasing the amount of primary amines appears to reduce transfection to hSkMC cells. This suggests that charged primary amines may prevent transfection to specific types of cell such as HskMC, which warrants further research to determine its importance for targeted delivery. As mentioned, comparing **P7** and **P10** shows the effect of block versus random for the glycosylated polymers. There is a clear difference here, with **P10** demonstrating a much higher transfection than **P7**. As well as this, reducing the charged block length from 10 (**P10**) to 5 (**P9**) caused a small drop in transfection. Overall, glycosylation appears to have the biggest impact on transfection as can be seen by **P8**, and there is some evidence to suggest that block polymers improve transfection over random polymers.

The last cell line explored was THP-1, a monocytic leukemia cell line that generally showed a significant immune response. Therefore, it was especially important to demonstrate promising transfection without drastically reducing the cell viability and interfering with their behavior. Moreover, THP-1 macrophages present carbohydrate-binding lectins and so should be sensitive to glycosylated polymers. In this cell line, polymers **P1**–**P7** performed similarly to saRNA. **P6** showed some better transfection efficiency compared to saRNA; however, there was significant error associated with the sample. Once again, **P8** showed optimum transfection efficiency, presumably due to the large amount of glycosylation being able to target the THP-1 cells. Nonetheless, the other glycosylated polymers **P7**, **P9**, and **P10** did not perform as well, although these polymers did not have as many glucose units attached. It must be noted that it is not clear whether the improved transfection is purely due to glycosylation or a synergy between the glycosylation and charged primary amines. Here, it would be of interest to synthesize more heavily glycosylated polymers, as there is a clear improvement demonstrated here by adding more glucose units.

## 4 Conclusions

In this study, the successful synthesis of charged and glycosylated poly(2-oxazoline)s was demonstrated. The synthesized polymers were combined with saRNA to form polyplexes, which were systematically tested for complexation efficiency, transfection in various cell lines, and cell viability. The polymer structure was shown to be influential over complexation efficiency of the saRNA, with block polymers showing complexation efficiencies of up to 95%. Furthermore, longer polymers also improved the complexation efficiency, and glycosylated polymers showed improved complexation compared to their nonglycosylated counterparts. Interestingly, **P8** which has the highest number of sugar units on the polymer chain generally had the best transfection efficiency in different cell lines, despite having an complexation efficiency of only 30%. The random glycosylated polymers **P7** and **P8** performed better in HEK 293 T/17 and HeLa cells, while **P8** outperformed all polymers in the hSkMC and THP-1 cell lines. Clearly the degree of glycosylation is a key factor in the transfection efficiency. While the difference between block and random polymers also influenced transfection, it was of secondary importance compared to glycosylation. Lastly, the polymers showed excellent cell viability across all cell lines and were comparable to lipofectamine. Future work in this area would be to increase the glycosylation of the polymers further and to maximize block lengths to increase transfection. Furthermore, evaluating the polymers in mixed cell cultures could be used to demonstrate targeted transfection in specific cell types.

## Supporting Information Available

Tables of DLS measurements for each polyplex at different N/P ratios, GPC traces of the synthesized polymers and ^1^H NMR of all prepared compounds (PDF)
