# Supplementary material for: Poly(2-oxazoline)/saRNA Polyplexes for Targeted and Nonviral Gene Delivery
Source: Biomacromolecules. 2023 Oct 4;24(11):5142–51. doi: 10.1021/acs.biomac.3c00683 (PMC10646937; doi:10.1021/acs.biomac.3c00683)
Supplement: Supplementary file 1 — bm3c00683_si_001.pdf [file bm3c00683_si_001.pdf]

Poly(2-oxazoline)/saRNA Polyplexes for Targeted and Non-viral Gene Delivery

Graham Hayes,[‡a] Beatriz Dias Barbieri,[‡b] Gokhan Yilmaz[a], Robin J. Shattock,[b*] and C. Remzi Becer[a*]

[a] Department of Chemistry, University of Warwick, Coventry, CV4 7AL, United Kingdom

[b] Department of Infectious Diseases, Imperial College London, Norfolk Place, London, W2 1PG, United Kingdom

E-mail: Remzi.becer@warwick.ac.uk and R.Shattock@imperial.ac.uk

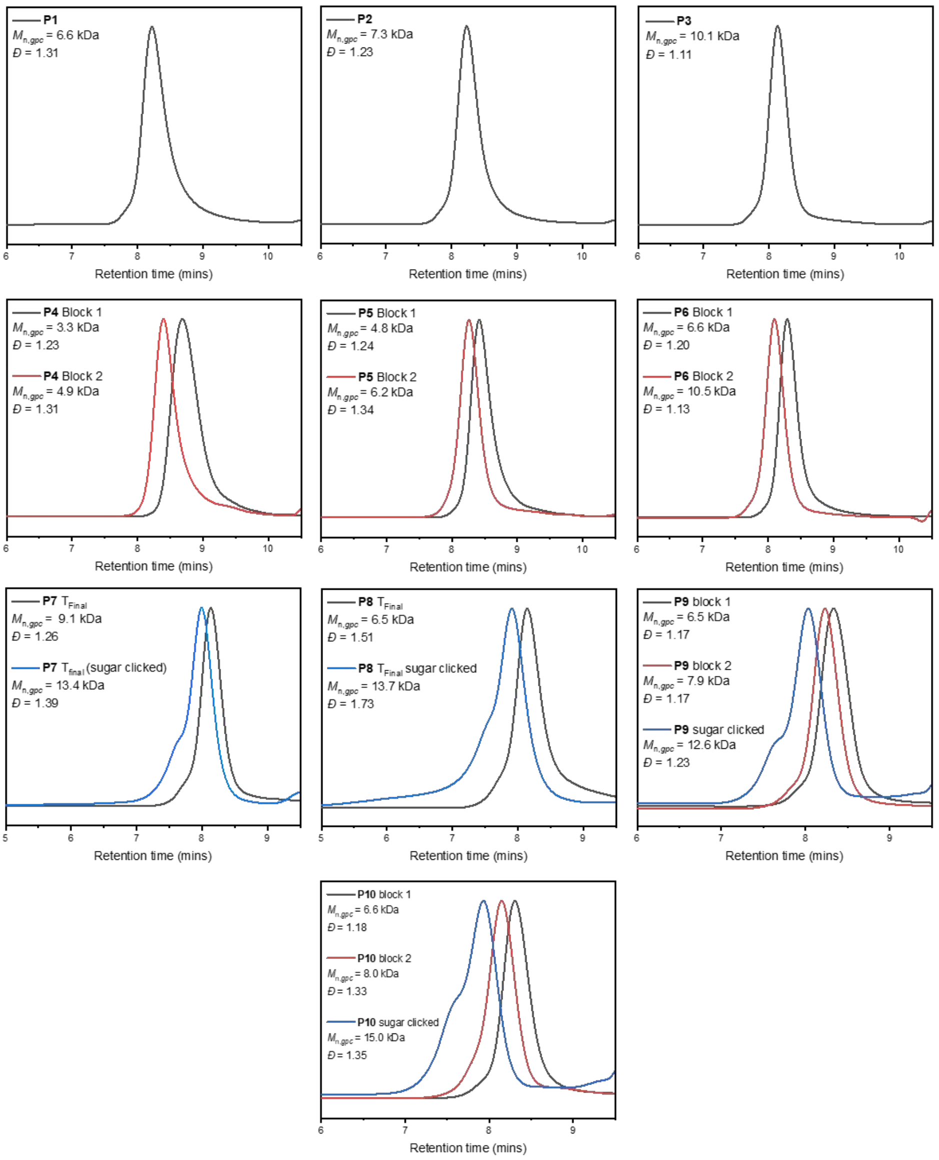

Figure S1. GPC traces of all polymers. Red traces indicate addition of a second block, blue traces indicated polymers after glycosylation.

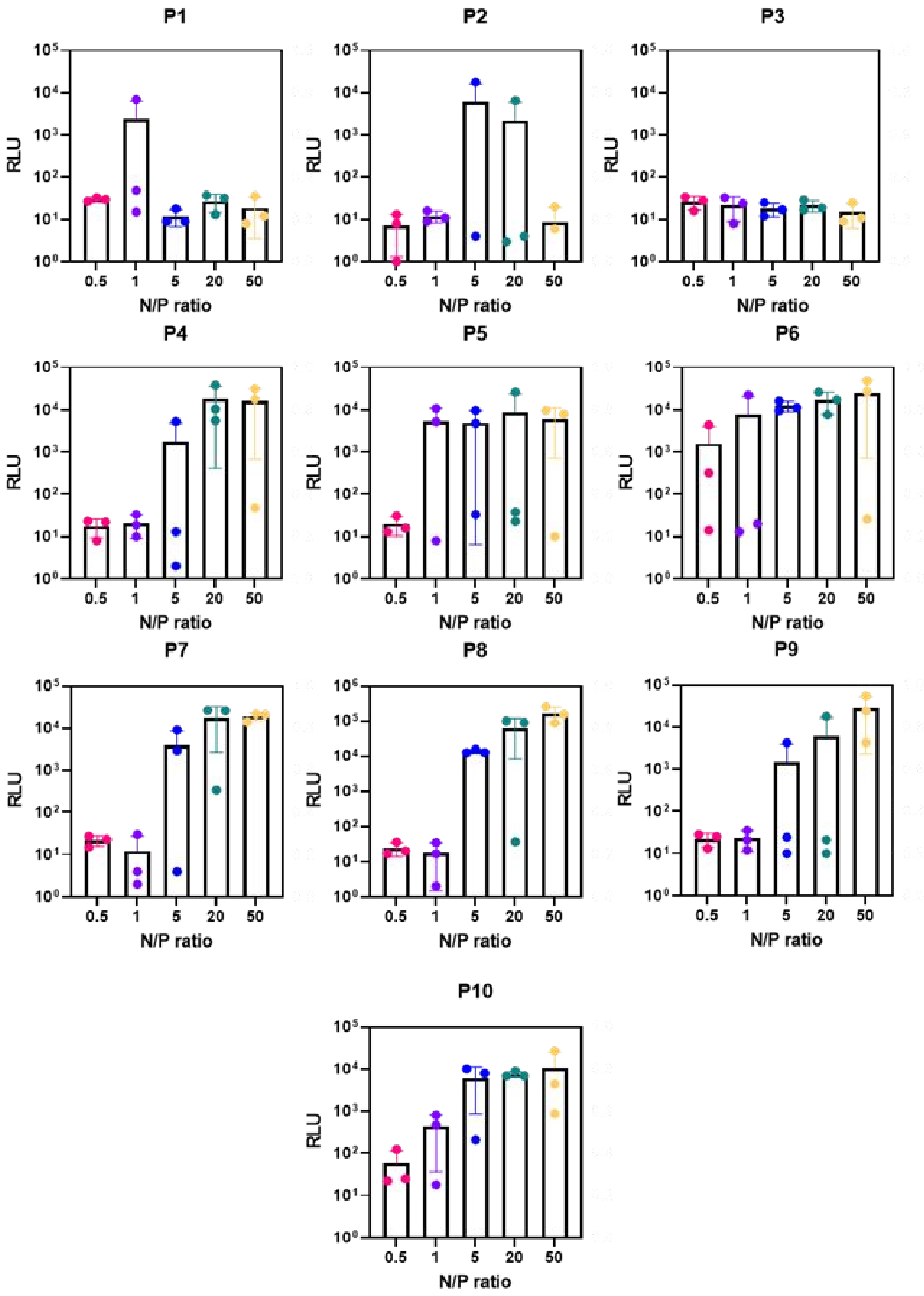

Figure S2. Transfection efficiencies for all polymers at N/P ratios of 0.5, 1, 5, 20, 50.

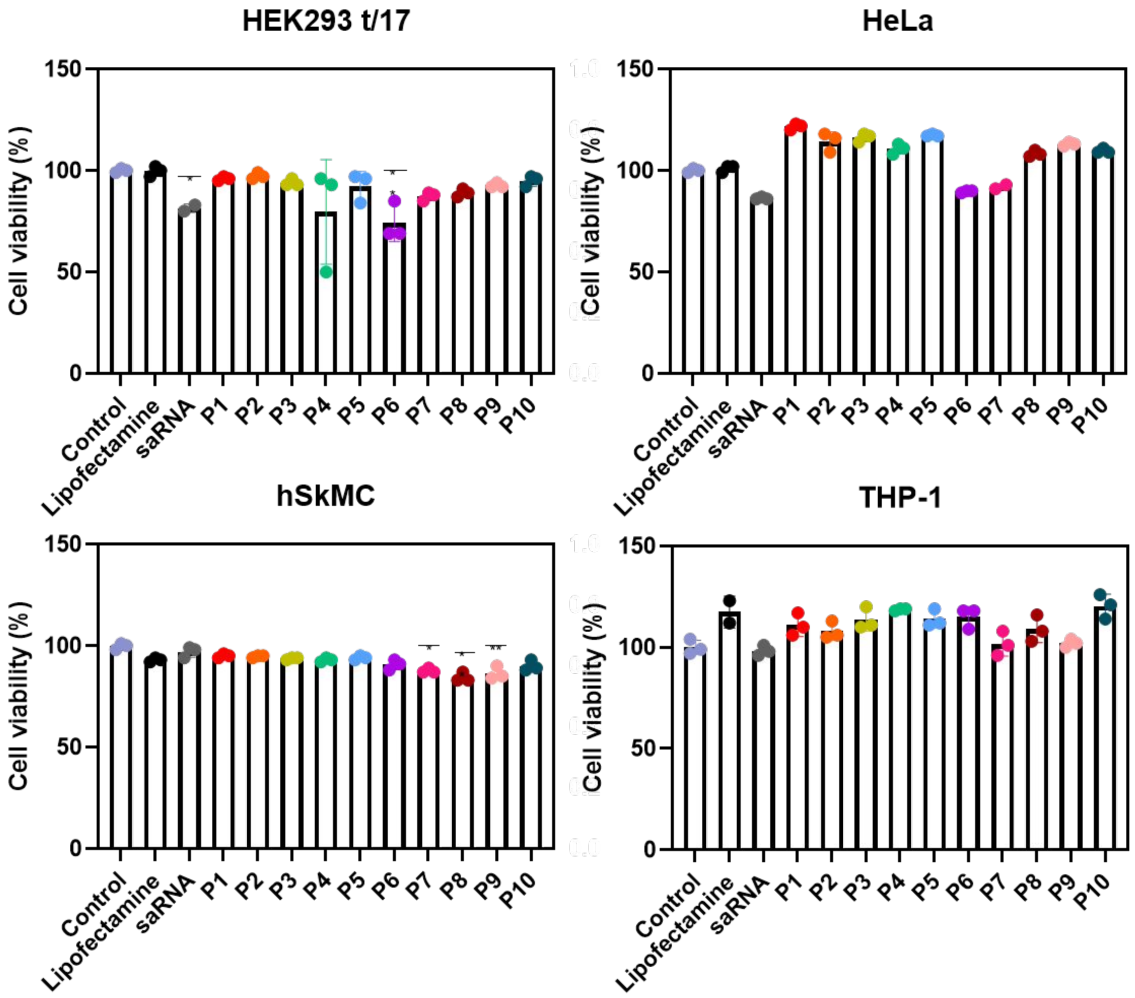

Figure S3. Cell viability of different cell lines upon incubation with the polymers for 24 hours, as calculated using the CellTiter-Glo 2.0 assay.

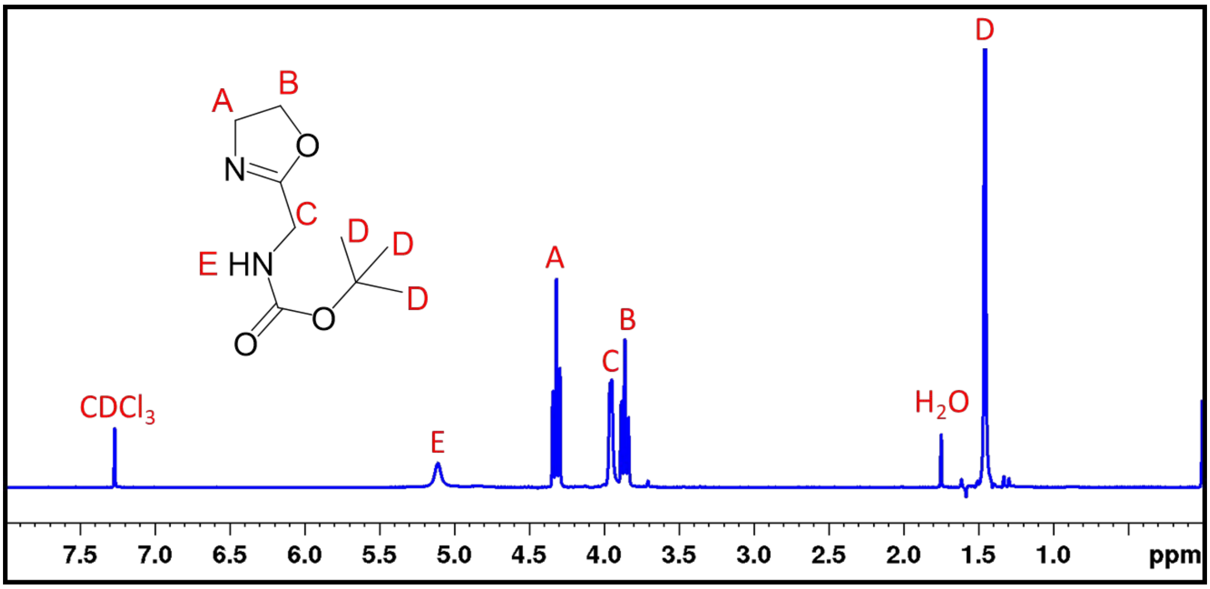

Figure S4. ^{1}H NMR Spectrum of BocOx.

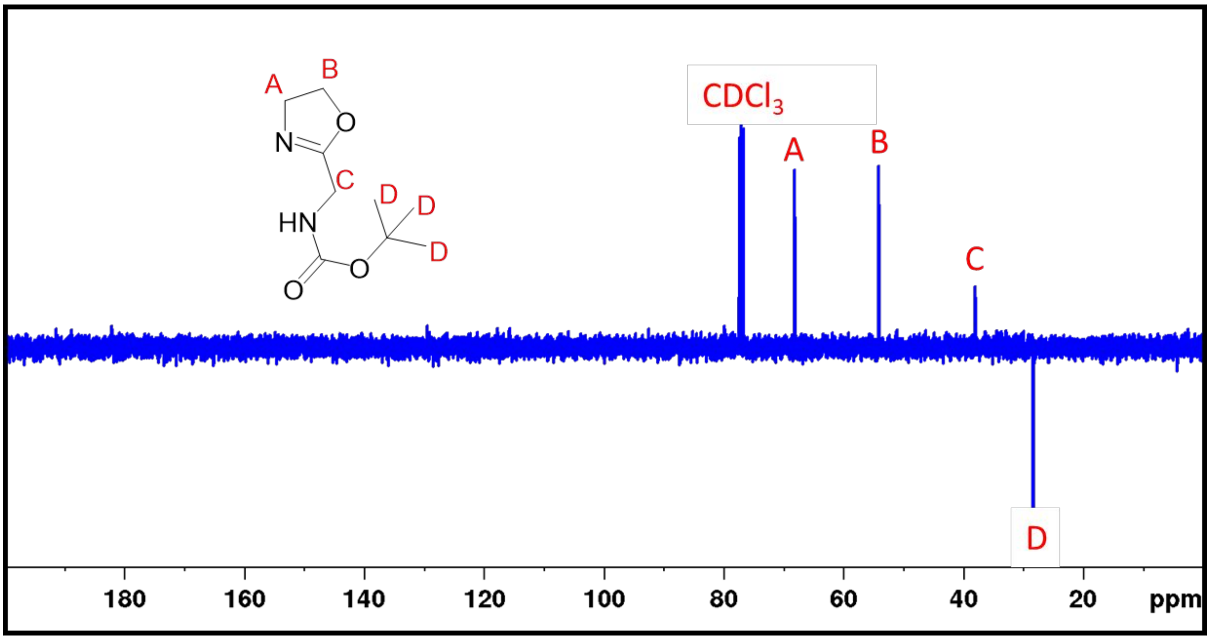

Figure S5. ^{13}C NMR of BocOx.

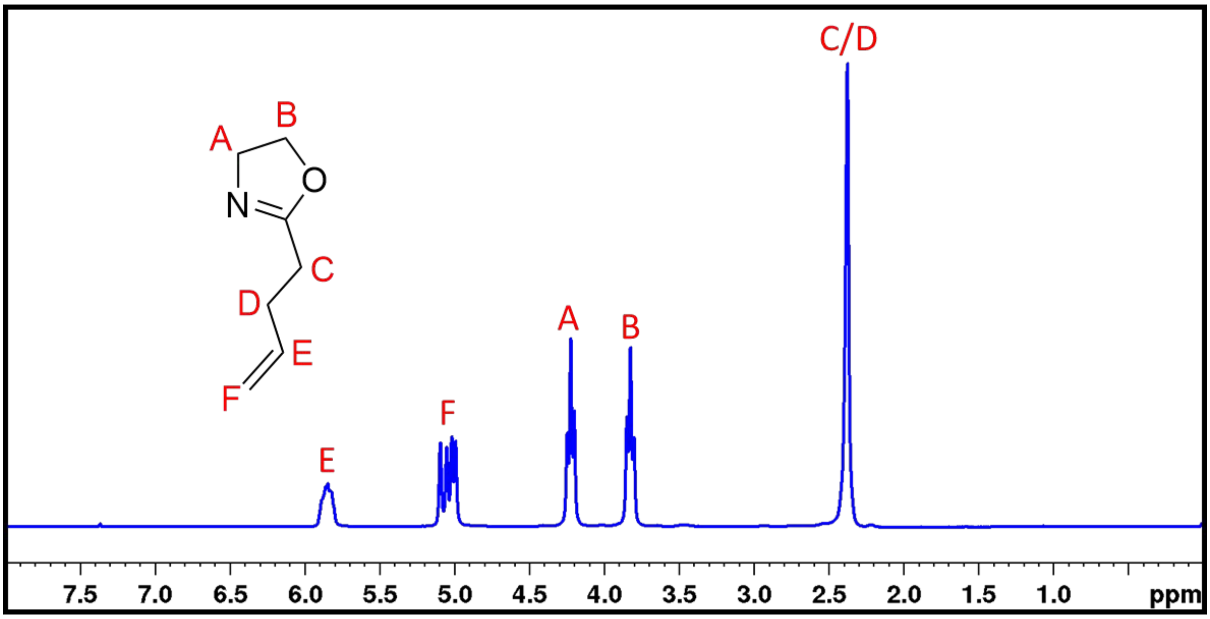

Figure S6. ^{1}H NMR of BocOx.

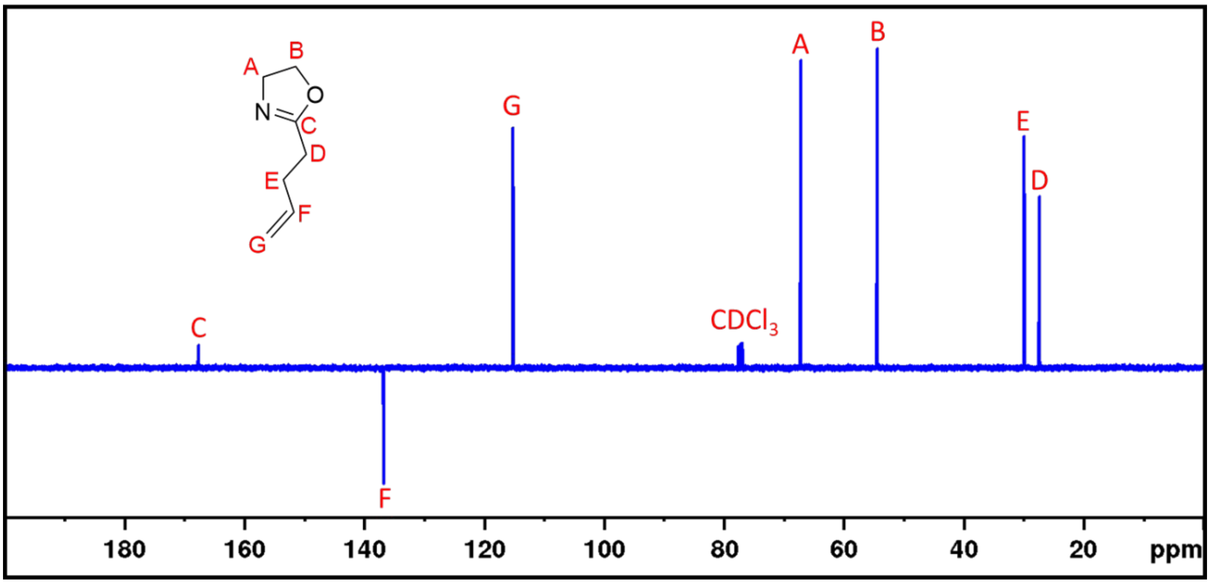

Figure S7. ^{13}C NMR of BocOx.

Table S1. Quantities and conditions for all polymers synthesised.

Polymer	*initiator*	*initiator*			*EtOx*			*BocOx*			*ButenylOx*			*concentration (M)*	*Reaction time (mins) (block 1)*	*Reaction time* (mins) (block 2)	*Reaction Temperature*
		mass (g)	*eqs*	*moles (mmol)*	*mass (g)*	*eqs*	*moles (mmol)*	*mass (g)*	*eqs*	*moles (mmol)*	*mass (g)*	*eqs*	*moles (mmol)*				
P1	PropTs	0.030	1	0.14	1.000	70	10.09	0.289	10	1.44	-	-	-	4	135	-	100
P2	PropTs	0.023	1	0.11	1.000	95	10.09	0.213	10	1.06	-	-	-	4	175	-	100
P3	PropTs	0.024	1	0.11	1.000	90	10.09	0.224	10	1.12	-	-	-	4	170	-	100
P4	PropTs	0.026	1	0.12	0.495	40	4.99	0.250	10	1.24	-	-	-	4	75	25	100
P5	PropTs	0.035	1	0.16	1.000	60	10.09	0.337	10	1.68	-	-	-	4	100	20	100
P6	PropTs	0.053	1	0.25	2.000	80	20.18	1.010	20	5.04	-	-	-	4	135	35	100
P7	MeOTs	0.015	1	0.08	0.395	50	3.99	0.160	10	0.80	0.100	10	0.80	4	100	-	100
P8	MeOTs	0.005	1	0.03	0.079	30	0.80	0.053	10	0.27	0.100	30	0.80	4	115	-	100
P9	MeOTs	0.015	1	0.08	0.395	50	3.99	0.16	10	0.80	0.100	10	0.80	4	100	20	100
P10	MeOTs	0.015	1	0.08	0.395	50	3.99	0.16	10	0.80	0.100	10	0.80	4	100	20	100

Table S2. Quantities of 1-Thio-β-D-glucose tetraacetate and DMPA used for P7-P10.

Polymer	*Polymer amount (mg)*			*double bonds per chain*	*mmoles of double bond*	*DMPA*			*sugar*		
	Mass (mg)	*RMM (g/mol)*	*moles (mol)*			*Eq*	*mmoles*	*mass (mg)*	*Eq*	*mmoles*	*Mass (mg)*
P7	100	12,000	8.3E-06	9	0.0001	0.5	0.04	10	5	0.375	138
P8	100	17700	5.6E-06	27	0.0002	0.5	0.08	20	5	0.763	278
P9	100	11100	9.0E-06	9	0.0001	0.5	0.04	10	5	0.405	147.5
P10	100	12200	8.2E-06	10	0.0001	0.5	0.04	11	5	0.410	150

Table S3. Size and PDI (as measured by DLS) and zeta potential for all polymers at N/P ratios of 0.5, 1, 5, 20, 50.

P1	Size (nm)	PDI	Zeta Potential (mV)
N/P 0.5	660 ± 906	0.81 ± 0.27	-3 ± 1
N/P 1	174 ± 43	0.52 ± 0.28	-2 ± 0
N/P 5	195 ± 28	0.39 ± 0.16	-4 ± 2
N/P 20	143 ± 33	0.55 ± 0.13	-3 ± 1
N/P 50	132 ± 16	0.47 ± 0.12	-7 ± 5

P2	Size (nm)	PDI	Zeta Potential (mV)
N/P 0.5	141 ± 57	0.85 ± 0.13	-10 ± 12
N/P 1	151 ± 23	0.55 ± 0.23	-6 ± 4
N/P 5	163 ± 31	0.74 ± 0.27	-14 ± 5
N/P 20	287 ± 77	0.60 ± 0.06	-10 ± 1
N/P 50	237 ± 49	0.74 ± 0.18	-14 ± 0

P3	Size (nm)	PDI	Zeta Potential (mV)
N/P 0.5	177 ± 14	0.72 ± 0.23	-5 ± 6
N/P 1	113 ± 25	0.93 ± 0.12	-2 ± 0
N/P 5	126 ± 16	0.51 ± 0.12	-5 ± 4
N/P 20	89 ± 10	0.63 ± 0.20	-8 ± 9
N/P 50	138 ± 18	0.39 ± 0.14	-6 ± 1

P4	Size (nm)	PDI	Zeta Potential (mV)
N/P 0.5	350 ± 62	0.40 ± 0.07	-18 ± 6
N/P 1	190 ± 14	0.35 ± 0.03	-21 ± 7
N/P 5	252 ± 24	0.34 ± 0.03	-10 ± 4
N/P 20	200 ± 41	0.31 ± 0.03	+4 ± 1
N/P 50	215 ± 47	0.40 ± 0.13	+5 ± 1

P5	Size (nm)	PDI	Zeta Potential (mV)
N/P 0.5	173 ± 14	0.33 ± 0.06	-3 ± 2
N/P 1	137 ± 10	0.40 ± 0.10	-4 ± 2
N/P 5	111 ± 7	0.44 ± 0.07	-3 ± 1
N/P 20	93 ± 4	0.33 ± 0.05	+4 ± 1
N/P 50	181 ± 40	0.43 ± 0.10	-2 ± 1

P6	Size (nm)	PDI	Zeta Potential (mV)
N/P 0.5	145 ± 22	0.37 ± 0.05	-10 ± 3
N/P 1	163 ± 5	0.24 ± 0.02	-51 ± 13
N/P 5	78 ± 4	0.27 ± 0.06	+1 ± 0
N/P 20	92 ± 13	0.43 ± 0.03	+5 ± 3
N/P 50	105 ± 8	0.54 ± 0.11	+2 ± 1

P7	Size (nm)	PDI	Zeta Potential (mV)
N/P 0.5	797 ± 233	0.59 ± 0.06	-32 ± 1
N/P 1	507 ± 69	0.58 ± 0.12	-24 ± 7
N/P 5	412 ± 40	0.47 ± 0.05	-19 ± 3
N/P 20	196 ± 6	0.20 ± 0.00	+9 ± 1
N/P 50	241 ± 7	0.42 ± 0.01	+8 ± 0

P8	Size (nm)	PDI	Zeta Potential (mV)
N/P 0.5	334 ± 38	0.45 ± 0.12	-23 ± 7
N/P 1	617 ± 75	0.51 ± 0.04	-19 ± 3
N/P 5	249 ± 24	0.51 ± 0.07	+2 ± 0
N/P 20	309 ± 43	0.54 ± 0.11	+14 ± 1
N/P 50	190 ± 38	0.83 ± 0.26	+10 ± 0

P9	Size (nm)	PDI	Zeta Potential (mV)
N/P 0.5	170 ± 95	0.43 ± 0.17	-15 ± 12
N/P 1	220 ± 29	0.40 ± 0.10	-14 ± 7
N/P 5	130 ± 6	0.42 ± 0.01	-15 ± 7
N/P 20	110 ± 28	0.39 ± 0.06	-8 ± 2
N/P 50	189 ± 43	0.42 ± 0.05	-5 ± 1

P10	Size (nm)	PDI	Zeta Potential (mV)
N/P 0.5	206 ± 42	0.36 ± 0.09	-11 ± 6
N/P 1	177 ± 34	0.34 ± 0.04	-17 ± 5
N/P 5	147 ± 14	0.49 ± 0.09	-15 ± 7
N/P 20	122 ± 2	0.43 ± 0.05	-4 ± 0
N/P 50	130 ± 6	0.41 ± 0.05	+1 ± 0

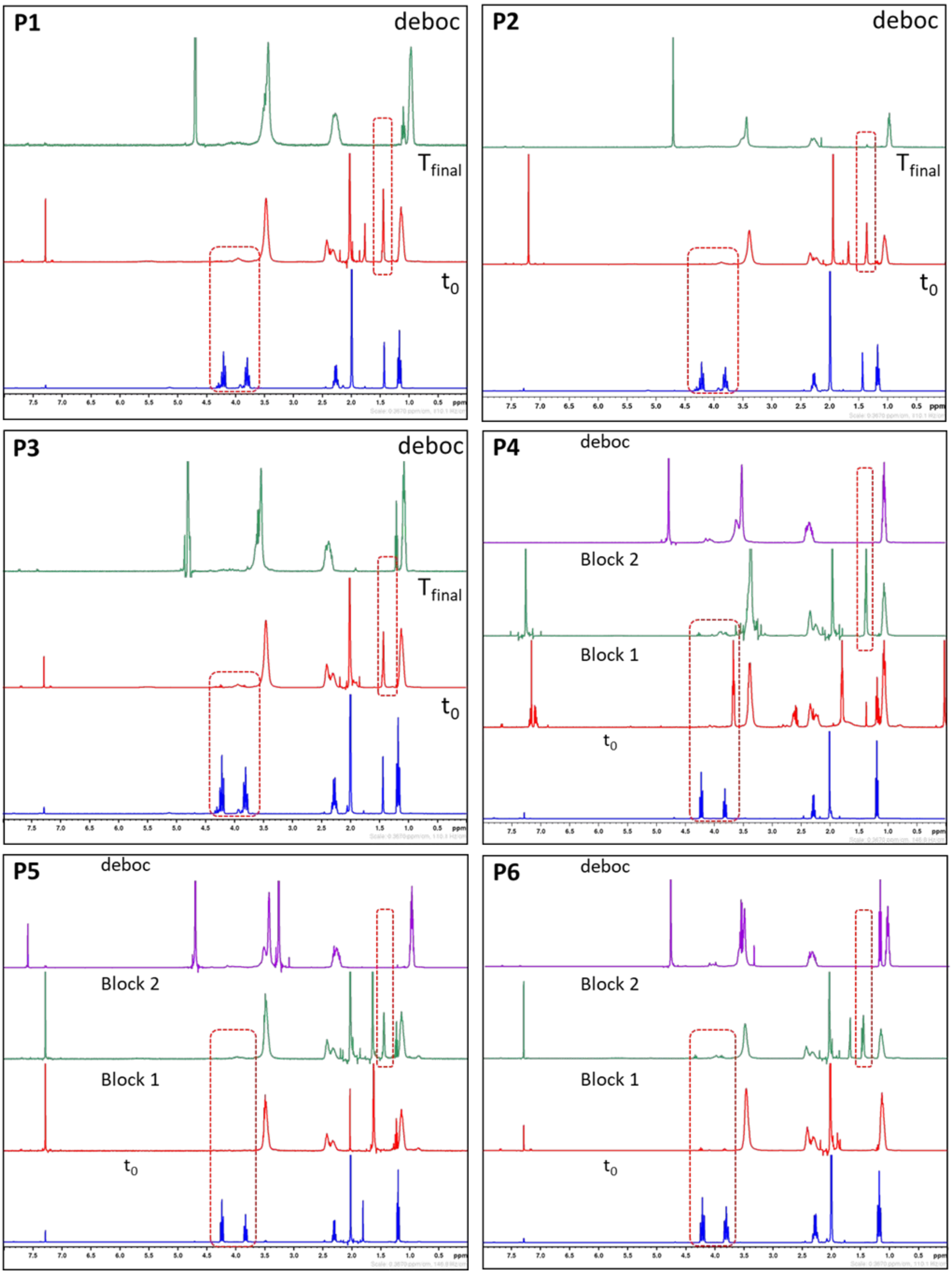

Figure S7. ^{1}H NMRs of each transformation for the non-glycosylated polymers **P1**-**P6.**

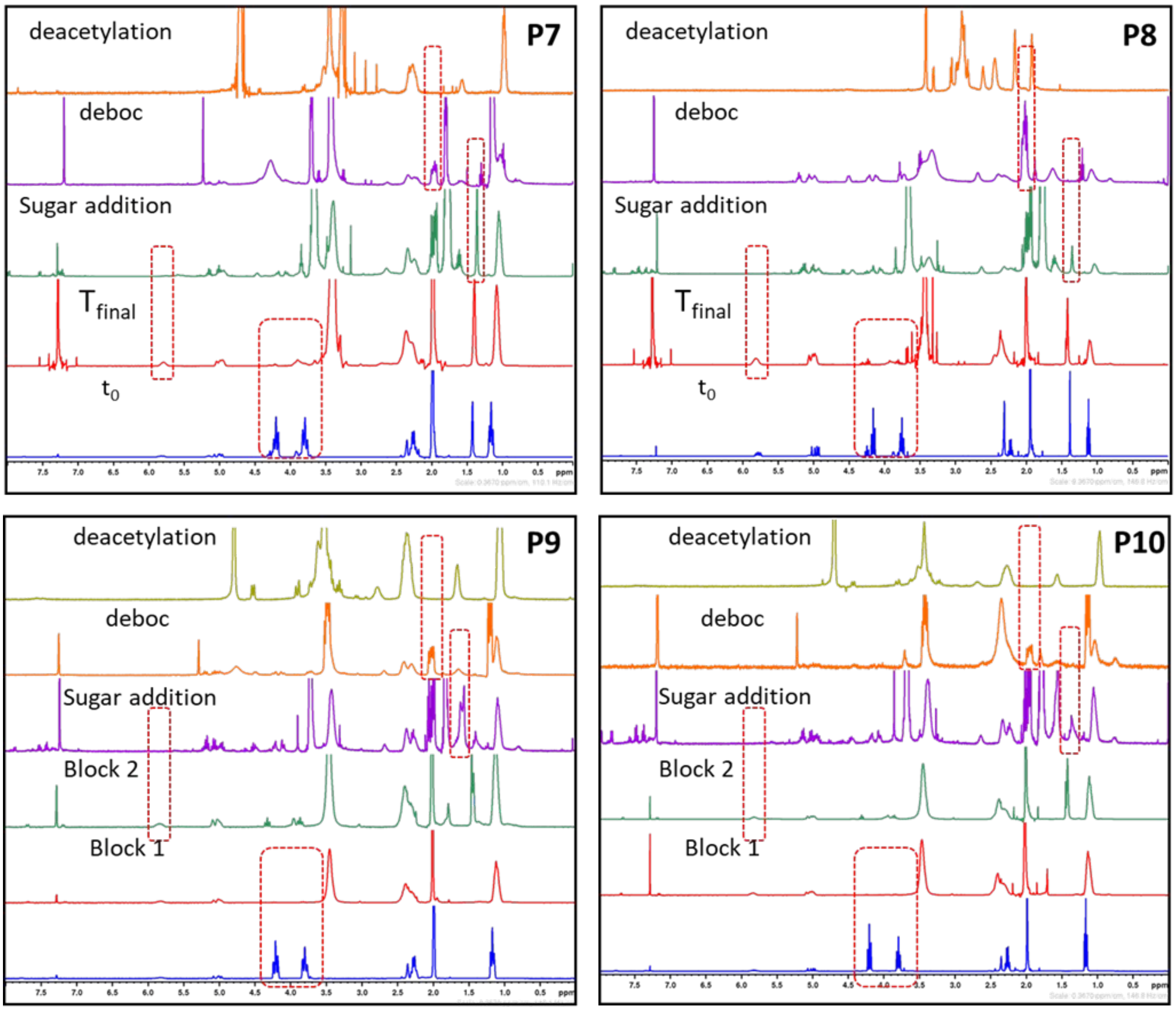

Figure S8. ^{1}H NMRs of each transformation for the glycosylated polymers **P7**-**P10.**
